# Impact of Recombinant Bovine Somatotropin on Bovine Milk Composition and *Fatty Acidome*: A Multidose Longitudinal Study

**DOI:** 10.3390/foods11213477

**Published:** 2022-11-02

**Authors:** Rocío Barreiro, Alexandre Lamas, José M. Miranda, Carlos M. Franco, Alberto Cepeda, Patricia Regal

**Affiliations:** Department of Analytical Chemistry, Nutrition and Bromatology, Faculty of Veterinary Science, Universidade de Santiago de Compostela, 27002 Lugo, Spain

**Keywords:** milk, bovine, quality, composition, minerals, fatty acid, rbST, somatotropin

## Abstract

Somatotropin is a species-specific polypeptide hormone produced in the pituitary gland of vertebrates. When administered exogenously to cattle, it can increase milk yield. However, the trade and administration of recombinant bovine somatotropin (rbST) to farm animals have been banned in the European Union (EU). Aside from food safety issues, very little is known about the effects of this hormone on milk composition and quality. In this work, a wide profile of fatty acids (the so-called *fatty acidome*) was determined by GC-FID in raw milk collected from control and rbST-treated lactating cows in a multidose longitudinal study. Milk composition (lactose, protein, fat, dry matter), including minerals (Ca, K, Mg, Na, P), was also determined, and milk yield was recorded. A tendency toward a less saturated profile was observed in the milk collected from animals treated with rbST, with higher concentrations of monounsaturated fatty acids. In addition, less calcium and potassium and more lactose and protein content were observed in milk from treated animals than in regular milk. As a result of this multicomponent profiling of milk, a clear impact of somatotropin treatment on milk quality was observed. The obtained results should be particularly interesting for those countries that permit the use of this hormone in dairy production.

## 1. Introduction

Growth hormone (GH), or somatotropin (ST), is a species-specific polypeptide hormone produced by the anterior lobe of the pituitary gland in vertebrates. It is a key regulator of somatic growth and is responsible for regulating several vital processes, including sexual maturation, immunity, and nutrient metabolism. The anabolic and growth effects of ST have led to different medical applications and improvement opportunities for the livestock industry [1]. After discovering that injecting pituitary extracts containing somatotropin into dairy cows could increase milk yield [2], the commercial synthesis of recombinant bovine ST (rbST) was initiated by Monsanto in 1993, enabling large-scale applications of this hormone and marking a major turning point in the history of the dairy industry. The administration of rbST has galactopoietic effects on lactating dairy cattle, as it both increases milk synthesis by the mammary glands and orchestrates other body processes to provide the nutrients required to support this rise in milk production, consistent with the concept of homeorhesis [3,4,5].

It soon became evident that rbST would be a historical milestone for dairy production, helping to satisfy the increasing worldwide demand for milk while reducing costs. rbST can be used to enhance milk production in cows and in other dairy ruminants, such as sheep, goats, and buffaloes [6]. rbST became a tool to increase profit as it happened to many other veterinary drugs, and not infrequently, it has been used with no consideration toward animal welfare [7,8]. However, consumers’ concerns related to food safety and animal welfare issues arose in parallel with the release of this drug into the market [8]. Studies suggest that rbST residues would remain in cow milk at ppb level no more than fourteen days after administration and that a blood-milk barrier specificity exists depending on the hormone formulation [9]. Upon ingestion, most of the potential rbST residues occurring in milk from treated animals would break down in the human digestive system. Even so, there are some concerns about the safety of these dairy products for consumers, in particular, related to increased insulin-like growth factor (IGF-1) levels, in comparison to non-treated cattle [10,11,12]. IGF-1 levels are not affected by pasteurization [13] or gastric fluids and it could play a role in human health [14]. Invoking these concerns and their impact on European milk policy, the European Union (EU) regulated the marketing and use of bovine somatotropin in its member states in 1999 through Council Decision 1999/879/EC [15]. Australia, Canada, Japan, and New Zealand prohibited its use and sale in their territories as well. Conversely, the administration of rbST to dairy animals is permitted in other countries, such as the USA, Brazil, and Mexico. In this context, several analytical methods have been developed to trace the presence of rbST in milk and other matrices and thus control its administration [16]. Confirmation of rbST (ab)use is not always easy since it requires tedious sample preparation, sophisticated analytical instruments, and very low detection limits. The use of natural-like variants further complicates the scenario in targeted methods. Alternative methods are always welcome, particularly to screen a large number of samples in short periods of time. Hence, untargeted and multianalyte approaches have also been investigated as alternatives to the challenging determination of rbST in biological matrices [10,17,18].

The great importance of milk and dairy not only for human nutrition but also to the economy of many countries is unquestionable. In this context, multiple milk parameters are monitored during its production and processing to assess its quality [19,20,21]. It is well accepted that the chemical composition of milk is substantially affected by a series of parameters, such as species, breed, lactation stage, animal age, health, feeding regime, and season. Considerable efforts have been invested in genomic breeding programs and the improvement of management systems focused on boosting bovine production performance (milk yield and composition, technological traits), health status, longevity, and animal reproduction, among others. Nevertheless, consumer perception of milk can be considered a major driver of dairy research, particularly regarding profiles of nutritionally relevant compounds contained in milk, such as fat and fatty acids, protein, antioxidants, and minerals. There has been a particular interest in comparing concentrations of saturated and unsaturated fatty acids under different production systems and environmental or physiological conditions [19,22,23,24]. It is clear that rbST is a cost-reducing technology for dairy farms [8] that allows spreading total costs over more milk production even with the additional inputs required to use the hormone. However, very little effort has been invested into defining the impact this treatment may have on the quality of the dairy products obtained from treated animals. In this sense, profiling and omics analytical technologies offer a good opportunity to assess various components simultaneously in food, covering both safety and nutritional issues.

This study is meant to profile the measurable effects of rbST administration on the compositional properties of bovine milk, including the targeted omics profiling of fatty acids (*fatty acidome*) and determination of physical-chemical quality (lactose, protein, fat, dry matter) and minerals. To this end, milk samples obtained from cows treated with biweekly doses of rbST over the course of eight months and milk samples from a control group of animals were analyzed. The potential of this approach as a screening strategy for rbST (ab)use in cattle has been explored.

## 2. Materials and Methods

### 2.1. Reagents and Chemicals

Methanol, sulfuric acid, isooctane, water, n-hexane, and anhydrous Na_2_SO_4_ were purchased from Merck (Darmstadt, Germany). Standard mixtures of fatty acid methyl esters “F.A.M.E. mix, C4:0 to C24:0” and “PUFA No. 1, marine source”, “Linoleic acid methyl ester, cis/trans-isomers” mixture, individual fatty acids (cis-9, trans-11 conjugated linoleic acid (CLA) and trans-10, cis-12 CLA isomers), and internal standard tricosanoic acid (C23:0) were obtained from Sigma Aldrich (Madrid, Spain). The standards were diluted in isooctane and calibrators in hexane for gas chromatography.

### 2.2. Animals and Samples

From a herd of dairy cows located in Lugo (Spain), a total of 9 Holstein cows were selected by expected calving, in an age range between 1.5 and 4 years and in the first or second lactation. These cows were housed separately on the same farm and always kept under real field conditions. The cows had ad libitum access to water and were fed twice a day under a regular dairy feeding regime. Six cows were selected to be treated with rbST (rbST group), and the other three were not treated (control group). The 9 animals were never separated and were milked twice a day in a separate milking parlor.

The rbST group was treated subcutaneously with 500 mg of rbST (Lactotropina, Elanco, Eli Lilly, Mexico) every 14 days. The first dose was administered when animals had been in lactation for 9–10 weeks (theoretical lactation peak), following the manufacturer’s instructions. A total of 12 doses were administered to each cow of the rbST group to cover practically the entire lactation period of the animals, except for one of the cows for whom the treatment was stopped 2 weeks earlier (11 doses) to observe the withdrawal effect. The time elapsed between the first and the last dose was 168 days. There was a 28-day gap between the 5th and sixth rbST doses (instead of 14 days) to observe if milk yield would drop accordingly (withdrawal effect). The total sample and data collection time in this study, including the time prior to the 1st dose and the time elapsed since the last dose, was 240 days. Milk was collected daily and analyzed the same day or after a maximum of 48 h of refrigeration at 8 °C. Milk production of each cow (L) was recorded every day of sample collection, and leftovers were discarded.

All procedures were performed after evaluation of the corresponding regional authorities (Service of Livestock Farming of the Provincial Government of Lugo, Regional Ministry of Rural Affairs, Galicia), and in accordance with EU guidelines on animal welfare. In particular article 1, point 5, of Directive 2010/63/EU of the European Parliament and of the Council on the protection of animals used for scientific purposes, shall apply to this study, and the animals described here are excluded from its scope.

### 2.3. Milk Fatty Acidome Analysis

Milk samples from forty different time points were analyzed to elucidate the fatty acid profile of milk over the whole study, including the following days: −7 days, 0 (predose sample at day 0, i.e., first treatment of the rbST group), 1–4, 7–11, 14, 16, 18, 21, 23, 28, 30, 32, 35, 37, 39, 42, 44, 46, 49, 51, 53, 57, 60, 63, 66, 73, 84, 88, 91, 122, 140, 168, 196 and 219 days.

The lipid extraction and determination of the fatty acid composition of raw milk samples were performed using the method proposed by Barreiro et al. in 2020 [25]. Briefly, 10 μL of milk was digested with 2 mL of H2SO_4_ in methanol (2.5%) overnight, and fatty acid methyl esters (FAMEs) were generated by methyl esterification in a water bath for 2 h at 60 °C. FAMEs were extracted from the aqueous phase with 1 mL of *n*-hexane and transferred to injection vials for their analysis. Gas chromatography (GC) coupled with flame ionization detection (FID) was used for the determination of fatty acids. Chromatographic analysis was performed with a 6850 GC system (Agilent Technologies, Palo Alto, CA, USA) equipped with a flame ionization detector (GC–FID) and a DB-Was capillary column (60 m, 0.25 mm id, 0.25 µm film thickness; Agilent Technologies, Inc., Santa Clara, CA, USA). Data were collected by the integrator Software GC ChemStation version B.03.02 (Agilent Technologies). All chromatograms were reviewed to check for proper peak integration of individual fatty acids by comparison of their relative retention times with those of FAME standards. FAME absolute peak areas were used to calculate experimental response factors and internal standards for response normalization, and results are reported as the weight percentage (% *w*/*w*) of total fatty acids.

Samples were analyzed in triplicate, and mean values were used for data analysis. A total of forty-two fatty acids (FAs) were identified. The total saturated fatty acids (SFAs) resulted from the sum of the individual saturated fatty acids: C6:0, C8:0, C10:0, C11:0, C12:0, C13:0, C14:0, C15:0, C16:0, C17:0, C18:0, C20:0, C22:0, and C24:0. For total monounsaturated fatty acids (MUFAs), the included fatty acids were C14:1n-5, C16:1n-9, C16:1n-7, C16:1n-5, C16:1n-13t, C17:1n-9, C18:1n-9, C18:1n-7, C20:1n-11, C20:1n-9, C22:1n-11, and C22:1n-9, and for total polyunsaturated fatty acids (PUFAs), the included fatty acids were C18:2n-6, C18:2n-6 9c-12t, C18:2n-6 9t-12c, CLA18:2n-7 9c-11t, CLA18:2n-6 10t-12c, C18:3n-6, C20:2n-6, C20:3n-6, C20:4n-6, C18:3n-3, C18:4n-3, C20:3n-3, C20:4n-3, and C20:5n-3, C22:5n-3, and C22:6n-3. Fatty acids are presented on a weight percentage basis as % w/w of all FAs.

### 2.4. Milk Gross Composition and Mineral Content

The milk samples selected for composition analysis were chosen representatively and randomly among those used for the fatty acid profiling. For gross composition, samples collected on the following days were analyzed: −18, −13, −6, 1, 8, 16, 23, 30, 37, 44, 51, 57, 66, 73, 84, 88 122, 140, 168, 196, and 219 days. For mineral determination, the following data points were included: −18, −13, −6, 1, 3, 6, 9, 11, and 12 days.

Fat, protein, lactose, and dry matter were measured using MilkoScan^TM^ 7RM at the Galician Interprofessional Laboratory of Milk (LIGAL) using the PE/LIGAL/34 protocol, developed according to ISO/IEC 17025:2017. Minerals (Ca, K, Mg, Na, and P) were determined by ICP-MS in the facilities of the Network of Infrastructures to Support Research and Technological Development (RIAIDT) of Universidade de Santiago de Compostela, as described by Sanjulián et al. in 2021 [26].

### 2.5. Milk Fat Lipid Quality Indexes

Various indexes of lipid quality were calculated from the data [24], including an index of atherogenicity (IA), index of thrombogenicity (IT), the ratio between hypocholesterolemic and hypercholesterolemic fatty acids (h/H), and the ratio between omega 6 and omega 3 fatty acids (ω-6/ω-3), as follows.

(a) Index of atherogenicity (IA): relationship between the sum of the main saturated fatty acids (considered pro-atherogenic) and the sum of the main classes of unsaturated (considered anti-atherogenic).

(1) IA=[C12:0+(4×C14:0)+C16:0]/[∑MUFA+∑PUFAn6+∑PUFAn3]

(b) Index of thrombogenicity (IT): relationship between prothrombogenetic (saturated) and antithrombogenic fatty acids (MUFAs, PUFAs n6, and PUFAs n3).

(2) IT=(C14:0+C16:0+C18:0)/(0.5×MUFA+0.5×PUFAn6+3×PUFAn3+PUFAn3/PUFAn6)

(c) Index ratio between hypocholesterolemic and hypercholesterolemic FAs (h/H)

(3) h/H=(C18:1n9+C18:2n6+C20:4n6+C18:3n3+C20:5n3+C22:5n3+C22:6n3)/(C14:0+C16:0)

(d) Ratio between omega 6 and omega 3 fatty acids (ω-6/ω-3)

### 2.6. Statistical Analysis

Statistical analyses were carried out in GraphPad Prism 9 (San Diego, CA, USA) and PASW Statistics 24.0 (SPSS Iberica, Madrid, Spain). For each variable, the assumption that the data followed a normal distribution was verified by means of the Shapiro-Wilk test, and to test for equality of variances, Levene’s test was applied. To evaluate the differences between the two groups, a t test was used. For nonparametric data, the Mann-Whitney U test was selected. Differences in milk fatty acids, gross composition, and minerals after rbST treatment were tested considering all control samples and all rbST samples in two single groups, regardless of time. Differences in milk yield between the two groups of animals were tested per week. Continuous data are expressed as the mean and standard deviation. Principal component analysis (PCA) with the standardized method and parallel analysis to select principal components were used to obtain a general overview of the variance among samples.

## 3. Results and Discussion

### 3.1. Effects of rbST on Bovine Milk Fatty Acidome

**Figure 1:**
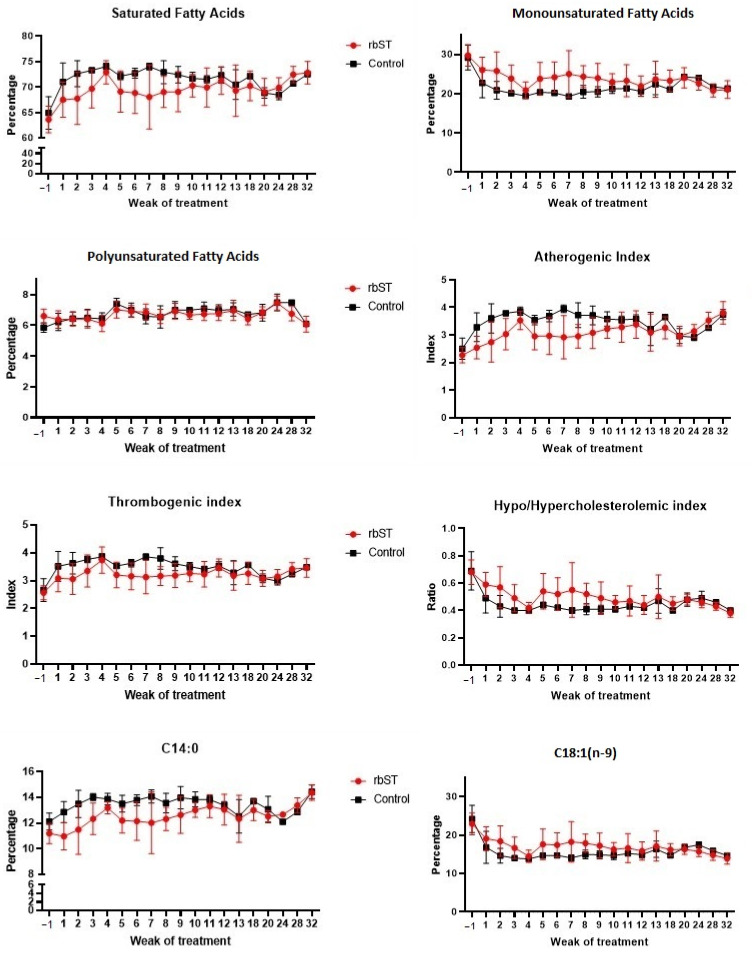
Evolution of various fatty acids (% *w*/*w* of total fatty acids) and quality indexes in milk from control and recombinant bovine somatotropin (rdST)cows presented over the course of 32 weeks of study (12 rbST biweekly doses).

The fatty acid profile of milk samples was determined in the control and rbST groups at different time points during the study, from one week before the treatment to 32 weeks after the first dose. No such long-term study has been performed thus far or published, and its value as an experiment representative of real farm conditions and covering the full lactation period shall be noted and considered in the field of rbST (a)buse. Data on SFAs are presented in Table 1, Table 2 and Table 3, MUFAs in Table 4 and Table 5, PUFAs in Table 6 and Table 7, fat fractions in Table 8, and indexes of lipid quality in Table 9. Considering the small sample sizes per day or week, and the uneven number of animals/samples (6 rbST cows, and 3 control animals) per group, inferential statistics for fatty acids were performed considering all control milk samples in one group and all milk samples collected after rbST treatment in another, with no consideration for sampling time. Not all data and/or individual fatty acids are shown in the tables due to space restrictions; values are presented as the mean and standard variation of the milk collected the same week. Figure 1 shows the evolution of different fatty acids and calculated indexes and ratios in the two groups of animals during the study, presented per week of treatment.

Some previous studies have found differences in the fatty acid profile of cows treated with rbST [27,28,29], especially a decrease in the levels of fatty acids C14:0 and an increase in the levels of C18:1. Eppard et al. (1985) observed that these modifications were dose-dependent [29]. In their study, they pointed out that significant differences were observed in the levels of C14:0 and C18:1 with the dose of 100 UI in comparison with the other doses evaluated (from 5 to 50 UI/dose). Similar modifications were observed in the present study (Table 1, Table 2, Table 3, Table 4, Table 5, Table 6 and Table 7). For inferential statistics, samples were considered as a whole, i.e., all milk samples collected after rbST administration were compared with samples obtained from animals with no treatment, independent of the time point. On the one hand, the percentage of C14:0 was significantly lower (*p* < 0.0001) in milk from rbST-treated animals (12.10 ± 1.54%) in comparison to control milk samples (13.40 ± 0.99%). In contrast to long-chain FAs and approximately half of C16:0, which are largely derived from the diet, the presence of C6:0 to C14:0 and a fraction of C4:0 in bovine milk is related to their synthesis *de novo* in the mammary gland [30]. The proportions of *de novo*-synthesized fatty acids increase with time during bovine lactation, and preformed fatty acids decrease in parallel. The administration of somatotropin can delay this shift in the origin of milk fatty acids [31], and for this reason, the percentage of C14:0 was lower in rbST samples. On the other hand, the percentage of C18:1 was significantly higher (*p* < 0.0001) in rbST milk (17.51 ± 3.50%) than in the control milk (15.63 ± 2.93%). Oleic acid is a predominant preformed fatty acid in adipocytes, and it is released during lipolysis. Not surprisingly, rbST induced a delay in the natural reduction of preformed FA over lactation, hence, C18:1 would be higher in milk from treated animals.

As for the fat fractions (Table 8), the rbST treatment caused a significant decrease (*p* < 0.0001) in the percentage of SFA and a significant increase (*p* < 0.0001) in MUFA, while it showed no effect (*p* = 0.64) on the percentages of polyunsaturated acids when comparing both control and rbST samples. Additionally, it is worth noting that the atherogenic index (AI) of milk fat (Table 9) was significantly lower (*p* < 0.0001) in rbST milk (2.81 ± 0.57) than in control samples (3.30 ± 0.48). Interestingly, the two previous findings could indicate a healthier profile of rbST milk fat for human consumption. The modifications were especially evident in the first weeks of treatment (Figure 1) and until week 10, and the differences decreased in the following weeks, as previously observed by Baer et al. (1989) [27].

**Figure 2:**
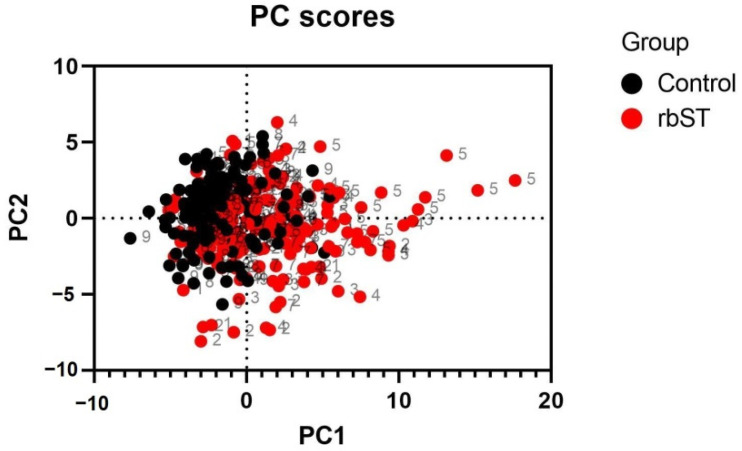
Principal component analysis scatter plot of the *fatty acidome* of bovine milk showing samples collected from control and recombinant bovine somatotropin animals at different time points during an experimental period of 240 days.

The obtained profiles at different time points during the whole study were submitted to principal component analysis (PCA), and the results are presented in Figure 2. These results demonstrated a change in the *fatty acidome* of cow milk associated with rbST treatments. The potential of this analytical technique for screening purposes should be further explored since *fatty acidomics* is a powerful straightforward approach that can be quickly implemented, at a relatively low cost, and following simple procedures. Milk is an undoubtedly useful matrix that can be easily collected daily, even twice a day, it is non-invasive, rich in fat, and sample processing is minimal. As such, it has already been pointed out as an ideal target sample for rbST monitoring [32]. However, it should be noted that fatty acids are involved in many biological processes and are influenced by many factors that cannot be controlled, so their profiles cannot be used for confirmation and must be considered carefully in the omics era. The previous limitations should be carefully considered when using omics or untargeted techniques to monitor hormone (ab)use in cattle, particularly in real conditions. For confirmation of rbST presence in milk, and hence, proof of its administration to the animal, methods based on liquid chromatography and mass spectrometry are available and described in the literature [33]. This approach is focused on detecting the tryptic N-terminal peptide, specific to the recombinant methionine-rbST form. Nonetheless, the limitations of using milk are well-known [32] and blood analysis by LC-MS/MS is recommended for enforcement purposes, in combination with screening strategies [9,34]. Plus, mass spectrometry enables the identification of alanine-rbST (Ala-rbST, identical to endogenous bST) and methionine-rbST (Met-rbST) variants. Both rbST formulations are traceable in serum at least 2 weeks after administration but Met-rbST is barely transferred to milk [9]. Additionally, both rbST alternatives induce similar levels of anti-rbST antibodies in serum and in milk.

### 3.2. Effects of rbST on Bovine Milk Gross Composition and Mineral Content

The physical-chemical composition and minerals of milk samples in the control and rbST groups at different time points during the study were determined. Minerals and gross composition (lactose, fat, protein, and dry matter or solids-nonfat) are presented in Table 10 and Table 11, respectively. Inferential statistics for composition and minerals were performed considering all control milk samples in one group and all milk samples collected after rbST treatment in another, regardless of sampling time. Milk yield over the course of the whole study is presented in Table 12, and differences between groups were tested per week. Not all data and/or individual days are shown in the tables due to space restrictions; values are presented as the mean and standard variation of the milk collected the same week.

For the minerals, considering samples as a whole, i.e., grouping all samples from treated animals and comparing them with milk from control individuals, the t test highlighted differences in Ca (*p* < 0.05) and in K (*p* < 0.001), meaning that milk from treated animals has lower levels of calcium and potassium. When analyzed by the week of treatment, the differences were not as evident but could still be seen (Table 10). Conversely, Eppard et al. in 1985 [29] concluded that exogenous growth hormone did not affect the concentration of calcium, phosphorus, sodium, iron, copper, and manganese in milk. However, Eppard’s samples were collected over a 10-day period of treatment, so the results are not comparable. In this work, the changes in mineral content are more evident later in time.

The physical-chemical quality of milk was measured using a standardized protocol for raw milk based on infrared spectroscopy (Fourier transform), including fat, protein, lactose, and dry matter. Considering samples as a whole, i.e., grouping all samples from treated animals and comparing them with milk from control individuals, the *t* test highlighted differences in lactose (*p* < 0.001), protein (*p* < 0.05), and dry matter (*p* < 0.001), meaning that milk from treated animals has higher levels. When analyzed by the week of treatment, the differences were not as evident but could still be seen (Table 11). The results agree with the data presented by Baer et al. in 1989 [27], who stated that milk serum protein and lactose were higher after somatotropin treatment. Similar conclusions were reached by De Morais et al. in 2017 using two rbST formulations [35], and by Molento et al. in 2002 [11] regarding protein content. The percentage of milk fat was not affected by treatment, as previously noted [35,36]. An increased dry matter intake has been related to rbST administration, and this fact could be related to the increased dry matter of milk too.

Table 12 presents a summary of the milk yield for the two groups of animals, expressed as the mean and standard deviation of their milk production (kg per day) at each week of the study, as well as the production difference between both groups (% of extra milk produced by the rbST group in comparison to the control group).

As reported in the work of Lamas et al. published in 2019, who applied transcriptomics to the milk somatic cells of these same 9 cows, the milk yield was highly variable among animals [17]. This fact was already expected, as real conditions were applied, and animals are naturally variable over their lactation cycle. As mentioned before, there was a 28-day gap between the fifth and sixth rbST doses of the 6 treated cows, plus one of those six animals received one dose less (the last dose on week 23 instead of 25). At week 16, one cow from the control group was retired from the study due to health issues. During the 28-day gap, a reduction in milk production in the rbST group can be observed, particularly in weeks 11 and 12, when biweekly treatment should have been administered. As soon as the rbST treatment was restored at week 13, the milk yield was recovered in the group. A maximum difference of 43% more milk in treated animals with respect to control cows was observed at cycle 12 by Lamas et al. [17]; a result both related to maintained production in one animal group due to rbST and a natural decreasing pattern in the other. Curiously, these significant differences were maintained until the end of the study, even almost two months after the last dose (12th dose, day 168) was administered. The most marked differences were observed between weeks 17 and 24, weakening at week 25. Curiously, that week is when the last dose was administered to the group, but not to all 6 of them but to 5 cows instead (however, the animal was considered in the same group for calculations).

## 4. Conclusions

The profiling of fatty acids in milk is an interesting alternative that can be used to assess the effects of rbST in animals and their products, as it is a simple, fast, and affordable technique. However, analysts should bear in mind that these molecules can be influenced by many factors, including pregnancy, feeding, or the health status of the cow. In this study, the existence of disturbance in bovine milk qualities upon treatment was confirmed, affecting the nutritional composition of the milk. The fat profile appears healthier, with less saturated and more monounsaturated fatty acids, while minerals are neglected. Lactose and protein showed an increasing tendency with rbST. Nonetheless, the number of animals included in this study is small, with only three control animals and six treated animals, and for this reason, the strength of the obtained results should be considered accordingly.

## Figures and Tables

**Table 1:** Short-chain and medium-chain saturated fatty acids in milk (% *w*/*w* of total fatty acids) obtained from cows treated with recombinant bovine somatotropin (rbST) (*n* = 6) and from control animals (*n* = 3) at different time points selected over the course of 37 weeks (8 injections of Lactotropina manufactured by Elanco, Eli Lilly, Mexico).

		C6:0				C8:0				C10:0				C11:0				C12:0			
Time		rbST		Control		rbST		Control		rbST		Control		rbST		Control		rbST		Control	
Days	Week	Mean	SD	Mean	SD	Mean	SD	Mean	SD	Mean	SD	Mean	SD	Mean	SD	Mean	SD	Mean	SD	Mean	SD
predose	−1	1.99	0.25	2.04	0.11	1.59	0.20	1.71	0.12	3.02	0.36	3.50	0.37	0.09	0.01	0.12	0.02	4.60	0.43	5.54	0.60
1 to 7	1	1.79	0.27	1.97	0.18	1.49	0.21	1.73	0.13	3.00	0.42	3.70	0.39	0.11	0.04	0.14	0.03	4.67	0.62	6.02	0.76
8 to 14	2	1.93	0.36	2.03	0.28	1.62	0.31	1.81	0.20	3.44	0.79	4.13	0.52	0.16	0.09	0.18	0.06	5.34	1.22	6.74	0.85
15 to 21	3	1.84	0.22	1.89	0.07	1.57	0.21	1.78	0.06	3.52	0.81	4.00	0.16	0.15	0.06	0.17	0.05	5.32	0.80	6.61	0.45
22 to 28	4	2.03	0.28	2.10	0.20	1.78	0.24	1.87	0.17	4.00	0.43	4.19	0.41	0.20	0.07	0.19	0.08	6.39	0.52	6.78	0.83
29 to 35	5	1.88	0.26	2.00	0.25	1.58	0.23	1.73	0.15	3.46	0.49	3.88	0.32	0.16	0.07	0.18	0.06	5.44	0.70	6.35	0.77
36 to 42	6	1.77	0.26	1.96	0.24	1.52	0.23	1.71	0.12	3.25	0.77	3.80	0.24	0.17	0.08	0.17	0.03	5.59	1.00	6.43	0.46
43 to 49	7	1.66	0.32	1.93	0.18	1.47	0.36	1.74	0.14	3.19	1.03	3.98	0.34	0.18	0.09	0.17	0.03	5.48	1.51	7.45	1.37
50 to 56	8	2.00	0.24	2.02	0.21	1.69	0.23	1.74	0.10	3.53	0.52	3.60	0.32	0.15	0.05	0.12	0.03	5.60	0.71	5.88	0.72
57 to 63	9	1.84	0.18	2.09	0.18	1.54	0.21	1.77	0.18	3.32	0.58	3.80	0.42	0.16	0.06	0.14	0.03	5.27	0.87	6.07	0.72
64 to 70	10	1.80	0.23	1.81	0.03	1.58	0.18	1.62	0.10	3.57	0.38	3.64	0.39	0.18	0.06	0.15	0.04	6.05	0.44	6.19	0.76
71 to 77	11	2.00	0.16	2.07	0.44	1.70	0.18	1.79	0.16	3.79	0.44	3.87	0.05	0.21	0.04	0.17	0.06	6.28	0.64	6.48	0.51
78 to 84	12	2.07	0.19	2.45	0.38	1.64	0.24	1.82	0.17	3.46	0.61	3.69	0.19	0.15	0.07	0.11	0.00	5.50	0.94	5.70	0.22
85 to 91	13	1.84	0.38	1.97	0.28	1.52	0.29	1.62	0.17	3.30	0.68	3.40	0.34	0.15	0.05	0.11	0.03	5.40	0.92	5.41	0.75
129 to 126	18	1.62	0.36	1.92	0.06	1.45	0.26	1.65	0.03	3.43	0.51	3.59	0.08	0.29	0.10	0.14	0.01	6.25	0.61	6.09	0.04
134 to 140	20	1.84	0.39	2.03	0.46	1.47	0.37	1.61	0.27	3.19	0.74	3.33	0.36	0.16	0.03	0.13	0.02	5.59	0.90	5.32	0.19
162 to 168	24	1.97	0.25	2.19	0.04	1.60	0.25	1.57	0.08	3.39	0.53	3.07	0.31	0.12	0.02	0.07	0.02	5.29	0.59	4.70	0.55
190 to 196	28	2.31	0.35	2.24	0.23	1.81	0.29	1.77	0.02	3.91	0.63	3.69	0.28	0.17	0.03	0.11	0.03	6.21	0.97	5.64	0.65
218 to 224	32	2.11	0.42	2.15	0.21	1.77	0.35	1.82	0.16	4.01	0.73	4.07	0.32	0.31	0.06	0.16	0.02	6.93	0.84	6.87	0.49

SD: Standard deviation.

**Table 2:** Long-chain saturated fatty acids (up to 17 carbons) in milk (% *w*/*w* of total fatty acids) obtained from cows treated with recombinant bovine somatotropin (rbST) (*n* = 6) and from control animals (*n* = 3) at different time points selected over the course of 37 weeks (8 injections of Lactotropina manufactured by Elanco, Eli Lilly, Mexico).

		C13:0				C14:0				C15:0				C16:0				C17:0			
Time		rbST		Control		rbST		Control		rbST		Control		rbST		Control		rbST		Control	
Days	Week	Mean	SD	Mean	SD	Mean	SD	Mean	SD	Mean	SD	Mean	SD	Mean	SD	Mean	SD	Mean	SD	Mean	SD
predose	−1	0.10	0.01	0.13	0.00	11.20	0.79	12.12	0.67	0.91	0.11	1.01	0.10	28.87	0.80	28.94	3.09	0.76	0.07	0.63	0.08
1 to 7	1	0.11	0.02	0.14	0.02	10.98	1.06	12.87	0.82	0.96	0.11	1.07	0.06	28.81	1.45	30.50	2.07	0.78	0.11	0.65	0.06
8 to 14	2	0.14	0.05	0.17	0.03	11.50	1.93	13.50	1.05	1.07	0.19	1.21	0.08	29.44	1.98	31.24	2.23	0.82	0.16	0.76	0.14
15 to 21	3	0.14	0.05	0.16	0.03	12.34	1.24	14.04	0.27	1.13	0.20	1.21	0.13	30.98	1.87	32.32	1.53	0.79	0.12	0.70	0.06
22 to 28	4	0.16	0.04	0.17	0.05	13.19	0.46	13.88	0.45	1.14	0.14	1.19	0.19	31.49	1.29	32.50	1.37	0.70	0.06	0.70	0.08
29 to 35	5	0.14	0.04	0.16	0.05	12.20	0.98	13.52	0.66	1.10	0.16	1.20	0.15	30.44	1.83	32.24	1.47	0.74	0.09	0.70	0.07
36 to 42	6	0.16	0.05	0.16	0.03	12.16	1.50	13.80	0.42	1.23	0.21	1.25	0.11	31.53	2.25	33.14	1.08	0.83	0.12	0.75	0.09
43 to 49	7	0.16	0.06	0.16	0.02	12.03	2.41	14.09	0.52	1.18	0.25	1.16	0.09	31.20	1.58	32.97	1.48	0.86	0.18	0.68	0.06
50 to 56	8	0.14	0.04	0.13	0.02	12.33	0.90	13.57	0.76	1.09	0.19	1.09	0.07	31.36	1.66	34.35	0.85	0.76	0.08	0.70	0.06
57 to 63	9	0.16	0.05	0.14	0.02	12.64	1.44	13.99	0.88	1.27	0.24	1.16	0.11	32.62	1.90	33.88	1.41	0.77	0.12	0.67	0.03
64 to 70	10	0.16	0.04	0.14	0.02	13.02	0.57	13.86	0.58	1.17	0.18	1.09	0.04	32.19	1.00	33.66	0.79	0.75	0.08	0.67	0.03
71 to 77	11	0.18	0.03	0.15	0.04	13.32	0.88	13.83	0.31	1.28	0.16	1.14	0.19	32.10	1.80	33.29	0.73	0.68	0.05	0.65	0.08
78 to 84	12	0.15	0.04	0.12	0.00	13.06	1.20	13.39	0.29	1.20	0.22	1.03	0.02	33.38	1.01	33.87	0.84	0.76	0.11	0.64	0.04
85 to 91	13	0.15	0.04	0.11	0.02	12.34	1.84	12.54	1.30	1.17	0.20	1.00	0.13	32.46	2.00	33.34	2.16	0.76	0.13	0.71	0.10
129 to 126	18	0.24	0.07	0.14	0.01	13.02	0.82	13.72	0.03	1.51	0.32	1.08	0.06	33.06	1.09	34.98	0.44	0.69	0.08	0.60	0.03
134 to 140	20	0.16	0.02	0.15	0.01	12.54	0.44	13.08	1.03	1.26	0.11	1.24	0.04	30.85	0.98	31.74	1.38	0.74	0.05	0.73	0.06
162 to 168	24	0.13	0.01	0.10	0.01	12.67	0.11	12.11	0.11	1.10	0.07	0.98	0.02	32.63	0.80	33.63	2.06	0.67	0.03	0.64	0.03
190 to 196	28	0.16	0.02	0.13	0.01	13.39	0.60	12.89	0.24	1.18	0.10	1.05	0.06	32.31	1.25	32.62	0.69	0.70	0.08	0.69	0.04
218 to 224	32	0.25	0.05	0.16	0.02	14.38	0.61	14.45	0.53	1.61	0.28	1.20	0.13	33.25	1.03	32.73	0.52	0.71	0.10	0.64	0.04

SD: Standard deviation.

**Table 3:** Long-chain C18:0 and very-long chain (≥ 22) saturated fatty acids in milk (% *w*/*w* of total fatty acids) obtained from cows treated with recombinant bovine somatotropin (rbST) (*n* = 6) and from control animals (*n* = 3) at different time points selected over the course of 37 weeks (8 injections of Lactotropina manufactured by Elanco, Eli Lilly, Mexico).

		C18:0				C20:0				C22:0				C24:0			
Time		rbST		Control		rbST		Control		rbST		Control		rbST		Control	
Days	Week	Mean	SD	Mean	SD	Mean	SD	Mean	SD	Mean	SD	Mean	SD	Mean	SD	Mean	SD
predose	−1	10.32	0.77	9.03	0.17	0.13	0.01	0.12	0.01	0.05	0.01	0.04	0.00	0.03	0.01	0.03	0.00
1 to 7	1	14.57	3.71	12.01	3.14	0.19	0.06	0.17	0.05	0.07	0.02	0.06	0.03	0.06	0.03	0.04	0.02
8 to 14	2	12.06	2.98	10.67	2.85	0.15	0.04	0.15	0.04	0.09	0.04	0.08	0.03	0.06	0.10	0.04	0.03
15 to 21	3	11.65	2.48	10.22	0.95	0.17	0.04	0.16	0.02	0.11	0.06	0.10	0.02	0.04	0.02	0.04	0.01
22 to 28	4	11.59	2.50	10.29	1.09	0.16	0.03	0.15	0.02	0.11	0.04	0.10	0.03	0.06	0.02	0.05	0.02
29 to 35	5	11.77	2.33	10.00	1.51	0.16	0.04	0.16	0.03	0.09	0.03	0.09	0.03	0.04	0.02	0.03	0.01
36 to 42	6	10.45	1.40	9.38	0.89	0.14	0.01	0.14	0.02	0.07	0.01	0.08	0.01	0.03	0.01	0.03	0.00
43 to 49	7	10.48	1.00	9.52	1.12	0.14	0.02	0.14	0.02	0.07	0.01	0.07	0.01	0.02	0.01	0.03	0.00
50 to 56	8	10.22	0.99	9.56	1.20	0.14	0.02	0.13	0.02	0.07	0.01	0.07	0.01	0.02	0.01	0.03	0.00
57 to 63	9	9.37	0.78	8.59	0.58	0.13	0.01	0.13	0.01	0.07	0.01	0.07	0.02	0.03	0.01	0.03	0.01
64 to 70	10	9.65	0.82	8.74	0.39	0.14	0.01	0.13	0.01	0.07	0.01	0.06	0.01	0.03	0.00	0.03	0.00
71 to 77	11	8.25	0.76	7.94	0.94	0.12	0.02	0.12	0.02	0.05	0.01	0.06	0.00	0.02	0.01	0.03	0.01
78 to 84	12	9.73	0.96	9.36	1.70	0.13	0.01	0.14	0.01	0.07	0.01	0.08	0.01	0.02	0.01	0.03	0.01
85 to 91	13	10.04	0.97	10.10	1.39	0.14	0.02	0.15	0.01	0.07	0.01	0.08	0.01	0.02	0.01	0.03	0.03
129 to 126	18	8.54	1.49	8.11	0.41	0.11	0.02	0.11	0.01	0.06	0.01	0.07	0.00	0.03	0.01	0.02	0.01
134 to 140	20	11.04	0.66	11.08	1.36	0.16	0.01	0.15	0.04	0.09	0.02	0.08	0.01	0.04	0.01	0.03	0.00
162 to 168	24	10.17	1.05	9.19	0.01	0.15	0.01	0.14	0.01	0.06	0.01	0.06	0.01	0.02	0.01	0.03	0.00
190 to 196	28	10.13	1.15	9.71	0.24	0.14	0.01	0.14	0.01	0.05	0.01	0.05	0.01	0.04	0.02	0.02	0.00
218 to 224	32	7.34	0.70	8.13	0.51	0.11	0.01	0.13	0.01	0.05	0.01	0.05	0.00	0.06	0.03	0.04	0.02

SD: Standard deviation.

**Table 4:** Monounsaturated fatty acids with 14, 16, or 17 carbons in milk (% *w*/*w* of total fatty acids) obtained from cows treated with recombinant bovine somatotropin (rbST) (*n* = 6) and from control animals (*n* = 3) at different time points selected over the course of 37 weeks (8 injections of Lactotropina manufactured by Elanco, Eli Lilly, Mexico).

		C14:1(n-5)				C16:1(n-9)				C16:1(n-7)				C16:1 (n-13)t				C17:1(n-9)			
Time		rbST		Control		rbST		Control		rbST		Control		rbST		Control		rbST		Control	
Days	Week	Mean	SD	Mean	SD	Mean	SD	Mean	SD	Mean	SD	Mean	SD	Mean	SD	Mean	SD	Mean	SD	Mean	SD
predose	−1	1.01	0.26	1.02	0.17	0.34	0.04	0.26	0.05	2.15	0.29	1.54	0.41	0.30	0.04	0.26	0.10	0.43	0.06	0.26	0.07
1 to 7	1	0.99	0.21	1.10	0.18	0.33	0.04	0.27	0.03	2.11	0.33	1.64	0.40	0.38	0.05	0.32	0.05	0.42	0.09	0.26	0.05
8 to 14	2	1.04	0.30	1.03	0.30	0.35	0.07	0.30	0.06	2.17	0.65	1.67	0.32	0.40	0.08	0.38	0.09	0.45	0.17	0.29	0.07
15 to 21	3	1.12	0.28	1.13	0.20	0.33	0.07	0.29	0.04	2.03	0.28	1.58	0.27	0.38	0.05	0.35	0.05	0.37	0.10	0.25	0.03
22 to 28	4	1.24	0.34	1.15	0.27	0.30	0.03	0.29	0.01	1.83	0.26	1.56	0.20	0.33	0.03	0.31	0.05	0.31	0.06	0.25	0.02
29 to 35	5	1.12	0.26	1.21	0.30	0.32	0.06	0.29	0.03	1.99	0.26	1.71	0.32	0.35	0.04	0.33	0.04	0.36	0.06	0.26	0.03
36 to 42	6	1.22	0.33	1.19	0.16	0.31	0.07	0.25	0.03	2.13	0.26	1.67	0.32	0.41	0.06	0.37	0.05	0.39	0.11	0.26	0.02
43 to 49	7	1.21	0.35	1.16	0.21	0.36	0.09	0.28	0.02	2.24	0.49	1.63	0.33	0.41	0.08	0.33	0.04	0.44	0.22	0.23	0.02
50 to 56	8	1.26	0.33	1.14	0.32	0.33	0.05	0.27	0.03	2.29	0.25	1.69	0.34	0.39	0.05	0.36	0.06	0.41	0.10	0.25	0.03
57 to 63	9	1.27	0.41	1.19	0.16	0.34	0.07	0.29	0.03	2.06	0.27	1.76	0.34	0.38	0.05	0.34	0.04	0.36	0.11	0.25	0.03
64 to 70	10	1.26	0.38	1.16	0.17	0.29	0.05	0.23	0.03	1.98	0.19	1.76	0.32	0.38	0.04	0.36	0.04	0.34	0.06	0.25	0.03
71 to 77	11	1.59	0.46	1.44	0.30	0.33	0.06	0.29	0.05	2.13	0.24	2.01	0.33	0.33	0.02	0.33	0.06	0.31	0.04	0.26	0.02
78 to 84	12	1.30	0.41	1.19	0.11	0.30	0.05	0.29	0.01	1.90	0.21	1.85	0.38	0.36	0.05	0.33	0.03	0.30	0.07	0.25	0.02
85 to 91	13	1.35	0.47	1.07	0.25	0.35	0.12	0.28	0.04	2.22	0.36	2.10	0.13	0.37	0.06	0.35	0.05	0.37	0.17	0.32	0.10
129 to 126	18	1.74	0.64	1.30	0.12	0.32	0.04	0.31	0.01	2.46	0.47	2.31	0.34	0.31	0.02	0.32	0.02	0.33	0.06	0.27	0.02
134 to 140	20	1.47	0.60	1.38	0.20	0.32	0.03	0.33	0.05	2.16	0.44	1.92	0.33	0.39	0.03	0.39	0.04	0.31	0.06	0.29	0.01
162 to 168	24	1.28	0.43	1.03	0.02	0.31	0.03	0.35	0.04	2.01	0.47	2.23	0.70	0.39	0.04	0.35	0.03	0.27	0.04	0.30	0.05
190 to 196	28	1.23	0.30	.95	0.18	0.32	0.03	0.33	0.02	1.76	0.25	1.85	0.23	0.36	0.03	0.35	0.03	0.26	0.03	0.26	0.04
218 to 224	32	1.96	0.47	1.52	0.33	0.32	0.06	0.30	0.06	2.45	0.26	2.42	0.24	0.34	0.03	0.32	0.03	0.33	0.08	0.30	0.05

SD: Standard deviation.

**Table 5:** Monounsaturated fatty acids with 18 carbons in milk (% *w*/*w* of total fatty acids) obtained from cows treated with recombinant bovine somatotropin (rbST) (*n* = 6) and from control animals (*n* = 3) at different time points selected over the course of 37 weeks (8 injections of Lactotropina manufactured by Elanco, Eli Lilly, Mexico).

		C18:1(n-9)				C18:1(n-7)				Isomers 18:1			
Time		rbST		Control		rbST		Control		rbST		Control	
Days	Week	Mean	SD	Mean	SD	Mean	SD	Mean	SD	Mean	SD	Mean	SD
predose	−1	22.91	2.84	24.20	3.54	0.72	0.05	0.56	0.12	1.46	0.46	0.80	0.25
1 to 7	1	19.08	3.09	16.81	4.14	0.70	0.07	0.60	0.06	1.58	0.33	1.33	0.32
8 to 14	2	18.43	3.98	14.63	1.93	0.71	0.08	0.65	0.05	1.77	0.30	1.52	0.15
15 to 21	3	16.67	2.79	14.01	0.43	0.68	0.08	0.59	0.05	1.77	0.48	1.46	0.29
22 to 28	4	14.51	1.59	13.82	0.63	0.58	0.05	0.57	0.03	1.39	0.46	1.11	0.40
29 to 35	5	17.61	3.99	14.68	0.59	0.66	0.06	0.62	0.03	0.97	0.16	0.89	0.10
36 to 42	6	17.43	3.16	14.72	0.51	0.67	0.09	0.61	0.03	1.20	0.64	0.78	0.11
43 to 49	7	18.23	5.25	14.08	0.72	0.67	0.12	0.58	0.04	0.99	0.32	0.69	0.04
50 to 56	8	17.89	2.39	14.95	1.13	0.65	0.08	0.60	0.07	0.72	0.07	0.83	0.39
57to63	9	17.24	3.30	14.88	1.06	0.66	0.09	0.62	0.04	1.21	0.57	0.77	0.06
64 to 70	10	16.29	1.77	14.68	1.05	0.65	0.04	0.66	0.06	1.37	0.39	1.72	0.10
71 to 77	11	16.62	3.76	15.22	0.46	0.61	0.06	0.62	0.04	0.99	0.37	0.81	0.02
78 to 84	12	15.89	2.39	14.93	0.89	0.61	0.07	0.60	0.02	0.82	0.05	0.79	0.00
85 to 91	13	17.15	3.93	16.32	2.14	0.61	0.10	0.59	0.12	0.80	0.11	0.94	0.51
129 to 126	18	16.19	1.63	14.75	0.32	0.65	0.05	0.58	0.00	0.89	0.16	0.87	0.04
134 to 140	20	16.33	1.35	16.81	0.73	0.66	0.03	0.70	0.05	1.94	0.46	1.91	0.13
162 to 168	24	15.74	1.36	17.47	0.83	0.70	0.06	0.72	0.00	1.43	0.66	1.16	0.37
190 to 196	28	14.80	1.37	15.93	0.13	0.65	0.06	0.72	0.04	0.90	0.11	0.95	0.01
218 to 224	32	13.89	1.46	14.68	0.53	0.56	0.05	0.59	0.00	0.75	0.09	0.73	0.02

SD: Standard deviation.

**Table 6:** Polyunsaturated fatty acids with 18 carbons in milk (% *w*/*w* of total fatty acids) obtained from cows treated with recombinant bovine somatotropin (rbST) (*n* = 6) and from control animals (*n* = 3) at different time points selected over the course of 37 weeks (8 injections of Lactotropina manufactured by Elanco, Eli Lilly, Mexico).

		C18:2(n-6)				C18:3(n-3)				C18:2(n-7)9,11t				C18:4(n-3)				C18:2(n-6)10t,12			
Time		rbST		Control		rbST		Control		rbST		Control		rbST		Control		rbST		Control	
Days	Week	Mean	SD	Mean	SD	Mean	SD	Mean	SD	Mean	SD	Mean	SD	Mean	SD	Mean	SD	Mean	SD	Mean	SD
predose	−1	3.41	0.37	2.95	0.06	0.54	0.07	0.54	0.03	1.52	0.28	1.43	0.22	0.10	0.03	0.08	0.02	0.10	0.02	0.10	0.02
1 to 7	1	3.23	0.35	3.13	0.20	0.51	0.07	0.57	0.06	1.41	0.27	1.48	0.26	0.10	0.03	0.09	0.03	0.12	0.04	0.11	0.02
8 to 14	2	3.38	0.28	3.39	0.24	0.55	0.06	0.61	0.05	1.29	0.22	1.33	0.26	0.13	0.06	0.10	0.04	0.13	0.03	0.13	0.02
15 to 21	3	3.44	0.31	3.40	0.19	0.55	0.08	0.58	0.03	1.22	0.18	1.33	0.38	0.15	0.07	0.13	0.06	0.17	0.04	0.14	0.03
22 to 28	4	3.38	0.37	3.58	0.25	0.54	0.07	0.60	0.04	1.06	0.18	1.10	0.16	0.11	0.06	0.10	0.01	0.12	0.06	0.17	0.04
29 to 35	5	3.90	0.28	4.09	0.18	0.65	0.08	0.74	0.03	1.28	0.20	1.39	0.28	0.14	0.03	0.16	0.03	0.19	0.07	0.20	0.05
36 to 42	6	3.78	0.21	3.83	0.24	0.61	0.07	0.68	0.04	1.23	0.23	1.30	0.22	0.13	0.03	0.12	0.03	0.16	0.03	0.16	0.03
43 to 49	7	3.80	0.31	3.60	0.23	0.58	0.09	0.60	0.05	1.16	0.27	1.21	0.26	0.12	0.03	0.12	0.02	0.18	0.04	0.17	0.04
50 to 56	8	3.54	0.24	3.51	0.39	0.55	0.07	0.59	0.10	1.17	0.25	1.22	0.27	0.12	0.04	0.11	0.02	0.17	0.04	0.20	0.05
57 to 63	9	3.70	0.27	3.73	0.29	0.62	0.07	0.64	0.05	1.28	0.24	1.31	0.19	0.14	0.03	0.15	0.04	0.19	0.04	0.23	0.05
64 to 70	10	3.40	0.20	3.61	0.03	0.57	0.08	0.64	0.05	1.20	0.20	1.40	0.13	0.16	0.02	0.14	0.03	0.22	0.04	0.18	0.03
71 to 77	11	3.51	0.18	3.72	0.23	0.59	0.09	0.63	0.07	1.31	0.20	1.40	0.20	0.13	0.02	0.14	0.02	0.20	0.02	0.20	0.04
78 to 84	12	3.57	0.26	3.65	0.14	0.62	0.07	0.64	0.07	1.18	0.24	1.27	0.21	0.13	0.03	0.13	0.03	0.21	0.04	0.23	0.04
85 to 91	13	3.58	0.39	3.69	0.34	0.64	0.06	0.66	0.10	1.20	0.24	1.15	0.16	0.18	0.07	0.17	0.04	0.24	0.05	0.22	0.04
129 to 126	18	3.32	0.23	3.49	0.16	0.51	0.06	0.56	0.02	1.14	0.16	1.29	0.09	0.17	0.03	0.16	0.03	0.23	0.03	0.20	0.00
134 to 140	20	3.51	0.18	3.34	0.39	0.52	0.06	0.47	0.06	1.12	0.12	1.22	0.07	0.18	0.03	0.21	0.06	0.24	0.04	0.22	0.03
162 to 168	24	3.67	0.25	3.67	0.52	0.74	0.09	0.73	0.05	1.43	0.12	1.47	0.05	0.18	0.05	0.19	0.02	0.28	0.04	0.27	0.04
190 to 196	28	3.48	0.38	3.82	0.21	0.68	0.10	0.75	0.02	1.10	0.10	1.49	0.13	0.14	0.04	0.13	0.01	0.23	0.05	0.23	0.00
218 to 224	32	3.04	0.22	3.05	0.06	0.61	0.06	0.61	0.03	0.99	0.15	1.14	0.17	0.12	0.03	0.11	0.02	0.20	0.03	0.19	0.01

SD: Standard deviation.

**Table 7:** Polyunsaturated fatty acids with 20 carbons in milk (% *w*/*w* of total fatty acids) obtained from cows treated with recombinant bovine somatotropin (rbST) (*n* = 6) and from control animals (*n* = 3) at different time points selected over the course of 37 weeks (8 injections of Lactotropina manufactured by Elanco, Eli Lilly, Mexico).

		C20:3(n-6)				C20:4(n-6)				C20:5(n-3)			
Time		rbST		Control		rbST		Control		rbST		Control	
Days	Week	Mean	SD	Mean	SD	Mean	SD	Mean	SD	Mean	SD	Mean	SD
predose	−1	0.17	0.02	0.13	0.03	0.23	0.03	0.20	0.03	0.06	0.04	0.04	0.00
1 to 7	1	0.18	0.03	0.15	0.03	0.24	0.04	0.21	0.02	0.10	0.05	0.08	0.04
8 to 14	2	0.18	0.02	0.17	0.03	0.23	0.03	0.21	0.07	0.06	0.02	0.06	0.02
15 to 21	3	0.19	0.04	0.17	0.03	0.25	0.04	0.23	0.01	0.06	0.02	0.06	0.02
22 to 28	4	0.19	0.04	0.17	0.03	0.29	0.05	0.24	0.02	0.10	0.05	0.05	0.03
29 to 35	5	0.21	0.04	0.18	0.02	0.26	0.04	0.26	0.05	0.09	0.06	0.06	0.01
36 to 42	6	0.20	0.02	0.17	0.01	0.28	0.04	0.24	0.02	0.06	0.01	0.05	0.01
43 to 49	7	0.20	0.04	0.17	0.01	0.29	0.07	0.25	0.03	0.06	0.01	0.06	0.02
50 to 56	8	0.21	0.05	0.18	0.03	0.29	0.02	0.27	0.03	0.06	0.01	0.06	0.01
57 to 63	9	0.18	0.03	0.15	0.02	0.26	0.03	0.25	0.03	0.07	0.01	0.07	0.02
64 to 70	10	0.19	0.04	0.17	0.01	0.27	0.02	0.25	0.04	0.08	0.01	0.09	0.02
71 to 77	11	0.18	0.03	0.15	0.00	0.27	0.03	0.28	0.06	0.05	0.01	0.07	0.02
78 to 84	12	0.20	0.04	0.15	0.03	0.28	0.03	0.24	0.06	0.06	0.01	0.09	0.02
85 to 91	13	0.20	0.04	0.16	0.03	0.28	0.05	0.29	0.04	0.07	0.01	0.09	0.04
129 to 126	18	0.16	0.04	0.14	0.03	0.22	0.04	0.24	0.02	0.09	0.02	0.07	0.01
134 to 140	20	0.20	0.03	0.19	0.03	0.28	0.02	0.27	0.02	0.09	0.02	0.11	0.03
162 to 168	24	0.17	0.03	0.15	0.03	0.23	0.05	0.24	0.01	0.07	0.01	0.08	0.00
190 to 196	28	0.19	0.05	0.17	0.05	0.21	0.03	0.23	0.04	0.07	0.02	0.06	0.01
218 to 224	32	0.17	0.03	0.16	0.04	0.23	0.02	0.22	0.02	0.07	0.01	0.06	0.01

SD: Standard deviation.

**Table 8:** Fat fractions in milk (% *w*/*w* of total fatty acids), i.e., saturated fatty acids (SFAs), monounsaturated fatty acids (MUFAs), polyunsaturated fatty acids (PUFA) omega 6 (w-6), and polyunsaturated fatty acids omega 3 (w-3) were obtained from cows treated with recombinant bovine somatotropin (rbST) (*n* = 6) and from control animals (*n* = 3) at different time points selected over the course of 37 weeks (8 injections of Lactotropina manufactured by Elanco, Eli Lilly, Mexico).

		SFA				MUFA				PUFA				PUFA w-3				PUFA w-6			
Time		rbST		Control		rbST		Control		rbST		Control		rbST		Control		rbST		Control	
Days	Week	Mean	SD	Mean	SD	Mean	SD	Mean	SD	Mean	SD	Mean	SD	Mean	SD	Mean	SD	Mean	SD	Mean	SD
predose	−1	63.61	2.62	64.92	3.20	29.74	2.74	29.19	3.16	6.60	0.46	5.85	0.31	0.85	0.06	0.78	0.02	4.13	0.35	3.55	0.13
1 to 7	1	67.51	3.44	71.01	3.72	26.04	3.28	22.71	3.78	6.38	0.57	6.22	0.56	0.86	0.10	0.85	0.11	3.99	0.35	3.78	0.24
8 to 14	2	67.74	5.11	72.63	2.58	25.77	4.88	20.86	2.24	6.40	0.44	6.44	0.44	0.87	0.10	0.90	0.09	4.12	0.31	4.08	0.31
15 to 21	3	69.64	3.77	73.31	0.44	23.84	3.44	20.11	0.65	6.41	0.59	6.48	0.56	0.84	0.19	0.90	0.11	4.20	0.34	4.10	0.24
22 to 28	4	72.90	2.28	74.05	0.66	20.87	2.11	19.42	0.46	6.12	0.53	6.43	0.38	0.82	0.13	0.88	0.10	4.15	0.34	4.29	0.23
29 to 35	5	69.11	4.08	72.15	0.72	23.78	4.15	20.37	0.66	7.01	0.54	7.40	0.37	0.94	0.11	1.00	0.07	4.63	0.31	4.81	0.15
36 to 42	6	68.83	4.03	72.72	0.99	24.19	3.86	20.20	0.63	6.91	0.38	7.00	0.45	0.94	0.07	0.98	0.05	4.58	0.22	4.56	0.27
43 to 49	7	68.06	6.32	74.02	0.65	25.02	5.96	19.33	0.65	6.86	0.53	6.58	0.49	0.89	0.13	0.87	0.09	4.62	0.30	4.33	0.25
50 to 56	8	69.01	3.06	72.91	2.26	24.35	2.77	20.47	1.63	6.57	0.47	6.54	0.73	0.86	0.07	0.88	0.11	4.36	0.28	4.24	0.45
57 to 63	9	69.07	3.91	72.44	1.62	23.95	3.75	20.49	1.32	6.92	0.52	7.00	0.55	0.97	0.11	0.99	0.09	4.48	0.32	4.46	0.36
64 to 70	10	70.29	2.21	71.71	1.25	22.98	2.11	21.24	1.21	6.67	0.28	6.99	0.05	1.00	0.11	1.00	0.03	4.24	0.26	4.40	0.08
71 to 77	11	69.93	3.86	71.52	0.73	23.29	4.06	21.34	0.36	6.73	0.42	7.08	0.43	0.93	0.12	1.01	0.04	4.28	0.21	4.47	0.21
78 to 84	12	71.24	2.71	72.34	1.61	21.91	2.60	20.60	1.27	6.77	0.47	6.98	0.36	0.98	0.04	1.11	0.08	4.40	0.24	4.37	0.21
85 to 91	13	69.30	5.07	70.49	2.92	23.67	4.58	22.39	2.64	6.96	0.66	7.04	0.44	1.10	0.10	1.16	0.12	4.42	0.45	4.52	0.37
129 to 126	18	70.23	2.92	72.14	0.11	23.30	2.70	21.08	0.14	6.41	0.38	6.71	0.03	1.01	0.12	1.01	0.04	4.04	0.26	4.21	0.10
134 to 140	20	69.02	2.66	68.83	1.04	24.05	2.57	24.27	0.50	6.84	0.37	6.82	0.55	1.10	0.07	1.20	0.11	4.38	0.18	4.18	0.39
162 to 168	24	69.90	1.95	68.43	0.94	22.58	1.70	24.03	0.38	7.45	0.45	7.49	0.55	1.30	0.12	1.29	0.06	4.45	0.24	4.46	0.52
190 to 196	28	72.46	1.60	70.69	0.02	20.74	1.58	21.77	0.04	6.75	0.47	7.49	0.01	1.19	0.07	1.20	0.01	4.23	0.36	4.58	0.13
218 to 224	32	72.83	2.20	72.54	0.51	21.05	2.25	21.32	0.55	6.07	0.51	6.09	0.04	1.09	0.17	1.02	0.02	3.80	0.25	3.74	0.14

SD: Standard deviation.

**Table 9:** Quality indexes of fat of milk obtained from cows treated with recombinant bovine somatotropin (rbST) (*n* = 6) and from control animals (*n* = 3) at different time points selected over the course of 37 weeks (8 injections of Lactotropina manufactured by Elanco, Eli Lilly, Mexico).

		w6/w3 ratio				CLAs				IA				TI				h/H			
Time		rbST		Control		rbST		Control		rbST		Control		rbST		Control		rbST		Control	
Days	Week	Mean	SD	Mean	SD	Mean	SD	Mean	SD	Mean	SD	Mean	SD	Mean	SD	Mean	SD	Mean	SD	Mean	SD
predose	−1	4.85	0.27	4.56	0.12	1.62	0.29	1.53	0.23	2.27	0.28	2.50	0.39	2.57	0.25	2.67	0.41	0.68	0.09	0.69	0.14
1 to 7	1	4.68	0.59	4.51	0.37	1.53	0.28	1.59	0.27	2.54	0.41	3.28	0.52	3.09	0.48	3.52	0.54	0.59	0.09	0.49	0.11
8 to 14	2	4.82	0.63	4.54	0.28	1.42	0.22	1.45	0.27	2.74	0.72	3.60	0.53	3.06	0.55	3.63	0.39	0.57	0.15	0.43	0.08
15 to 21	3	5.22	1.18	4.59	0.44	1.38	0.19	1.48	0.37	3.03	0.57	3.79	0.08	3.35	0.58	3.77	0.12	0.49	0.10	0.40	0.01
22 to 28	4	5.14	0.82	4.95	0.56	1.14	0.20	1.26	0.18	3.53	0.34	3.86	0.15	3.74	0.47	3.86	0.11	0.42	0.04	0.40	0.02
29 to 35	5	4.99	0.46	4.83	0.32	1.45	0.24	1.58	0.30	2.95	0.49	3.54	0.17	3.21	0.46	3.53	0.05	0.54	0.13	0.44	0.02
36 to 42	6	4.90	0.40	4.64	0.26	1.39	0.24	1.46	0.23	2.97	0.68	3.69	0.21	3.16	0.48	3.63	0.14	0.52	0.12	0.42	0.02
43 to 49	7	5.28	0.75	4.98	0.34	1.34	0.30	1.38	0.26	2.92	0.79	3.95	0.14	3.13	0.60	3.86	0.10	0.55	0.20	0.40	0.02
50 to 56	8	5.11	0.50	4.83	0.30	1.35	0.29	1.42	0.28	2.95	0.43	3.72	0.45	3.17	0.34	3.80	0.39	0.52	0.08	0.41	0.04
57 to 63	9	4.64	0.52	4.50	0.35	1.46	0.27	1.54	0.23	3.08	0.57	3.71	0.34	3.19	0.43	3.61	0.25	0.49	0.12	0.41	0.03
64 to 70	10	4.26	0.22	4.40	0.10	1.42	0.19	1.59	0.10	3.22	0.34	3.58	0.26	3.27	0.30	3.51	0.15	0.46	0.05	0.41	0.02
71 to 77	11	4.63	0.48	4.44	0.25	1.51	0.23	1.60	0.20	3.28	0.55	3.55	0.14	3.23	0.46	3.41	0.11	0.47	0.11	0.43	0.02
78 to 84	12	4.52	0.17	3.96	0.44	1.39	0.28	1.51	0.25	3.38	0.50	3.58	0.15	3.46	0.32	3.53	0.15	0.44	0.07	0.42	0.01
85 to 91	13	4.05	0.66	3.91	0.22	1.44	0.27	1.37	0.17	3.09	0.67	3.22	0.60	3.18	0.52	3.29	0.43	0.50	0.16	0.47	0.09
129 to 126	18	4.04	0.39	4.17	0.25	1.37	0.17	1.49	0.09	3.26	0.40	3.65	0.02	3.26	0.39	3.57	0.00	0.45	0.05	0.40	0.01
134 to 140	20	4.01	0.22	3.49	0.23	1.36	0.14	1.44	0.09	2.96	0.35	2.96	0.27	3.08	0.29	3.09	0.16	0.48	0.04	0.48	0.05
162 to 168	24	3.44	0.28	3.48	0.57	1.71	0.15	1.74	0.09	3.14	0.24	2.92	0.12	3.15	0.25	2.99	0.14	0.46	0.04	0.49	0.05
190 to 196	28	3.55	0.29	3.82	0.15	1.33	0.12	1.72	0.13	3.53	0.29	3.26	0.02	3.42	0.23	3.24	0.05	0.43	0.03	0.46	0.01
218 to 224	32	3.53	0.34	3.68	0.22	1.19	0.17	1.33	0.15	3.80	0.41	3.74	0.18	3.46	0.33	3.49	0.02	0.38	0.03	0.40	0.01

CLAs: Conjugated linoleic acid as % *w*/*w* of total fatty acids; h/H: Index ratio between hypocholesterolemic and hypercholesterolemic fatty acids; IA: Index of atherogenicity; SD: Standard deviation; TI: Index of thrombogenicity.

**Table 10:** Minerals of milk obtained from cows treated with recombinant bovine somatotropin (rbST) (*n* = 6) and from control animals (*n* = 3) at different time points selected over the course of 37 weeks (8 injections of Lactotropina manufactured by Elanco, Eli Lilly, Mexico).

		Ca (mg kg^−1^)				K (mg kg^−1^)				Mg (mg kg^−1^)				Na (mg kg^−1^)				P (mg kg^−1^)			
Time		rbST		Control		rbST		Control		rbST		Control		rbST		Control		rbST		Control	
Days	Week	Mean	SD	Mean	SD	Mean	SD	Mean	SD	Mean	SD	Mean	SD	Mean	SD	Mean	SD	Mean	SD	Mean	SD
−6	−1	1050.1	76.1	1062.3	48.3	1506.9	57.3	1497.3	58.2	111.2	4.2	96.7	12.9	350.6	30.9	371.6	128.4	1320.3	133.4	1173.9	115.0
1	1	1075.3	78.4	1082.4	32.7	1527.7	80.2	1538.1	54.9	111.4	5.0	96.4	13.4	342.6	17.5	337.9	61.9	978.4	78.2	898.8	24.7
17	3	997.3	66.9	1066.6	79.2	1404.7	113.3	1513.6	57.2	110.1	4.0	102.5	17.9	368.7	19.7	328.7	38.8	1231.0	99.0	1194.9	126.2
37	6	1094.1	67.8	1212.2	104.2	1539.5	114.1	1626.4	86.1	112.0	6.0	108.7	16.8	331.2	28.0	326.1	82.9	1010.8	93.1	1029.4	46.6
60	9	977.2	85.3	1040.5	120.6	1390.3	79.8	1440.8	80.9	104.9	6.1	104.8	25.1	365.5	52.6	360.3	84.8	1163.6	129.9	1159.1	203.6
73	11	1007.6	125.9	1105.7	98.8	1413.5	71.7	1450.9	20.5	105.2	10.3	109.6	23.4	358.4	33.5	357.1	33.6	1206.7	104.3	1207.7	158.0
84	12	1018.1	65.3	1090.9	92.1	1437.3	98.4	1445.3	34.4	106.5	5.0	109.2	17.5	378.7	103.4	381.0	79.1	1168.0	94.9	1186.5	84.0

SD: Standard deviation.

**Table 11:** Composition of milk obtained from cows treated with recombinant bovine somatotropin (rbST) (*n* = 6) and from control animals (*n* = 3) at different time points selected over the course of 37 weeks (8 injections of Lactotropina manufactured by Elanco, Eli Lilly, Mexico).

	Lactose (g/100 g)				Fat (g/100 g)				Protein (g/100 g)				Solids-nonfat (g/100 g)			
Time	rbST		Control		rbST		Control		rbST		Control		rbST		Control	
Week	Mean	SD	Mean	SD	Mean	SD	Mean	SD	Mean	SD	Mean	SD	Mean	SD	Mean	SD
−1	4.83	0.13	4.74	0.31	3.48	0.44	2.93	1.06	2.88	0.05	2.89	0.14	8.47	0.12	8.36	0.40
1	4.88	0.13	4.72	0.18	2.56	0.46	2.69	0.41	2.87	0.08	2.77	0.24	8.54	0.18	8.22	0.40
2	4.94	0.22	4.69	0.17	3.30	0.72	3.78	0.63	2.95	0.10	2.92	0.15	8.61	0.24	8.28	0.31
3	4.98	0.13	4.87	0.23	1.86	0.71	2.22	0.67	3.03	0.10	3.06	0.17	8.80	0.12	8.72	0.38
4	4.95	0.18	4.93	0.25	2.32	1.02	2.05	0.72	3.13	0.12	3.05	0.13	8.91	0.20	8.76	0.39
5	4.92	0.16	4.87	0.22	2.40	1.17	2.16	0.76	3.07	0.14	3.00	0.13	8.80	0.17	8.61	0.35
6	4.86	0.13	4.84	0.23	3.36	0.36	3.08	0.31	3.15	0.11	3.05	0.08	8.78	0.11	8.61	0.29
7	4.92	0.12	4.87	0.23	3.46	0.73	3.01	0.34	3.09	0.19	3.05	0.09	8.71	0.15	8.59	0.27
8	4.87	0.14	4.80	0.30	3.58	0.94	3.03	0.39	3.12	0.23	2.96	0.14	8.67	0.27	8.43	0.43
10	4.83	0.17	4.75	0.25	3.35	0.41	3.03	0.14	3.16	0.14	2.98	0.05	8.69	0.12	8.42	0.30
11	4.87	0.15	4.87	0.22	3.32	0.27	3.25	0.20	3.29	0.20	3.14	0.13	8.80	0.17	8.62	0.38
12	4.89	0.19	4.81	0.14	3.37	0.47	3.22	0.10	3.20	0.25	3.08	0.13	8.73	0.28	8.51	0.31
13	4.79	0.21	4.79	0.14	3.87	1.09	3.49	0.18	3.22	0.14	3.03	0.20	8.68	0.23	8.47	0.32
18	4.83	0.10	4.87	0.23	3.37	0.65	3.20	0.06	3.46	0.12	3.21	0.07	8.99	0.09	8.77	0.32
20	5.45	0.18	5.23	0.15	2.30	0.57	3.24	0.35	3.93	0.27	3.49	0.12	10.21	0.42	9.52	0.29
24	4.71	0.18	4.79	0.13	3.43	0.34	3.43	0.01	3.26	0.15	3.07	0.19	8.68	0.12	8.56	0.40
32	4.81	0.14	4.91	0.22	3.45	0.53	3.41	0.04	3.61	0.26	3.39	0.11	9.12	0.27	9.00	0.37

SD: Standard deviation.

**Table 12:** Milk yield of the group of cows treated with recombinant bovine somatotropin (rbST) (*n* = 6) and control (*n* = 3) animals, over the course of 37 weeks (8 injections).

Time *	Milk Production (L/day)				Difference (L/day)
	rbST Cows		Control Cows		Control Versus rbST
Weeks	Mean	SD	Mean	SD	Mean
−1	40.2	5.9	33.5	10.7	**−6.7**
1	40.9	6.5	35.7	10.3	**−5.2**
2 †	40.2	6.9	35.2	9.7	**−5.0**
3	41.6	6.0	37.3	8.8	**−4.3**
4	42.9	5.6	37.5	9.2	**−5.4**
5	43.0	5.1	37.9	8.5	**−5.1**
6	41.6	6.5	37.8	7.7	**−3.8**
7	40.5	9.5	37.4	6.9	**−3.0**
8	36.6	6.8	32.7	6.3	**−3.9**
9	37.2	8.5	34.9	7.2	**−2.4**
10	37.2	7.7	34.7	6.6	**−2.6**
11	36.0	7.2	34.1	4.9	**−1.8**
12	34.0	6.5	32.3	4.5	**−1.7**
13 †	36.5	8.6	30.5	5.5	**−6.0**
14 †	33.7	9.7	26.4	7.4	**−7.3**
15	32.2	8.3	27.1	5.3	**−5.1**
16 †	33.5	7.6	28.0	3.5	**−5.6**
17 ††	35.6	8.1	28.2	2.3	**−7.4**
18 ††	37.0	6.7	30.3	3.8	**−6.7**
19 ††	36.0	6.3	27.2	2.3	**−8.8**
20 ††	37.8	6.3	29.4	2.9	**−8.5**
21 ††	35.4	6.0	26.7	1.8	**−8.7**
22 ††	34.0	6.1	28.2	2.8	**−5.8**
23 ††	33.3	5.1	23.8	6.3	**−9.6**
24 ††	30.8	5.7	21.4	7.4	**−9.4**
25	34.7	6.3	25.0	6.1	**−9.7**
26 †	29.9	7.3	24.5	3.6	**−5.4**
27 †	29.3	5.3	24.9	3.1	**−4.4**
28 †	29.8	5.0	25.3	5.0	**−4.5**
29	30.7	4.8	27.5	4.3	**−3.3**
30 ††	28.9	4.9	24.4	2.4	**−4.6**
31	28.1	5.1	24.5	3.2	**−3.6**
32 †	28.7	4.7	23.8	7.2	**−4.9**
33 †	27.8	4.8	24.8	2.2	**−3.0**
34 †	27.7	4.9	23.9	2.5	**−3.8**

* Timeline relative to the 1st dose/injection of rbST (Lactotropina, Elanco) as day 0. At week 11 (day 70), the injection of rbST was omitted, at week 13 (day 84) restarted; at week 25 (day 168) the final/12th dose was administered, except for 1 animal (11th dose, last at day 154, week 23). † Significant differences in milk production between groups at *p* < 0.01 †† Significant differences in milk production between groups at *p* < 0.001.

## Data Availability

The data presented in this study are available on request from the corresponding author.
